# Diversity of *Meq* gene from clinical Marek’s disease virus infection in Saudi Arabia

**DOI:** 10.14202/vetworld.2016.572-578

**Published:** 2016-06-10

**Authors:** Mahmoud H. A. Mohamed, Ibrahim M. El-Sabagh, Malik A. Al-Habeeb, Yousef M. Al-Hammady

**Affiliations:** 1 Department of Clinical Studies, College of Veterinary Medicine, King Faisal University, Al-Hufof, 31982, Saudi Arabia; 2 Department of Avian and Rabbit Medicine, Faculty of Veterinary Medicine, Zagazig University, 44519, Zagazig, Egypt; 3 Central Biotechnology Laboratory, College of Veterinary Medicine, King Faisal University, Al-Hufof, 31982, Saudi Arabia; 4 Department of Virology, Faculty of Veterinary Medicine, Cairo University, 12211, Giza, Egypt; 5 Excutive Department of Risk Assessment, Saudi Food and Drug Authority, Saudi Arabia; 6 Central Veterinary Laboratory, Riyadh, Saudi Arabia

**Keywords:** deletion, Marek’s disease virus, *Meq* gene sequence, phylogenic analysis, Saudi Arabia

## Abstract

**Aim:**

The aim of this study was to demonstrate the genomic features of *Meq* gene of Marek’s disease virus (MDV) recently circulating in Saudi Arabia (SA).

**Materials and Methods:**

Two poultry flocks suffering from mortalities and visceral tumors were presented to the Veterinary Teaching Hospital, King Faisal University, SA. Subjected to different diagnostic procedures: Case history, clinical signs, and necropsy as well as polymerase chain reaction followed by *Meq* gene sequence analysis.

**Results:**

Case history, clinical signs, and necropsy were suggestive of MDV infection. The *Meq* gene was successfully detected in liver and spleen of infected chickens. A 1062 bp band including the native *Meq* ORF in addition to a 939 bp of S-Meq (short isoform of *Meq*) were amplified from Saudi 01-13 and Saudi 02-13, respectively. The nucleotide and deduced amino acids sequences of the amplified *Meq* genes of both Saudi isolates showed distinct polymorphism when compared with the standard USA virulent isolates Md5 and GA. The sequence analysis of the *S-Meq* gene showed a 123 bp deletion representing 41 amino acids between two proline-rich areas without any frameshift. The *Meq* gene encoded four repeats of proline-rich repeats (PRRs sequences), whereas the *S-Meq* contains only two PRRs. Interestingly, the phylogenetic analysis revealed that both of SA MDV isolates are closely related to the MDV strains from Poland.

**Conclusion:**

The two MDV isolates contain several nucleotide polymorphisms resulting in distinct amino acid substitutions. It is suggested that migratory and wild birds, as well as world trading of poultry and its by-products, have a great contribution in the transmission of MDVs overseas.

## Introduction

Marek’s disease (MD) is lymphoproliferative disease of chickens caused by the highly infectious cell-associated alphaherpesvirus MD virus serotype 1 (MDV1) or Gallid herpesvirus 2 and induces malignant lymphomas in chickens [1]. Currently, MD has been effectively controlled using the vaccines along with good management practice, and major losses to the poultry industry as a result of the disease have largely been averted [2-4].

The MDV genome of Md5 strain is about 177,874 bp linear dsDNA; it is predicted to encode 103 proteins [5]. The genetic basis and molecular mechanisms underlying viral virulence and oncogenicity remain poorly understood. The search for viral factors related to oncogenicity identified the viral genes encoding proteins involved in T-cell transformation (*Meq*) and others with potential involvement in tumorigenicity, viral virulence, and host range (pp24, pp38, viral interleukin 8) [6,7].

The *Meq* gene encodes a 339-amino acid protein with an N-terminal basic region leucine zipper (bZIP) domain and a C-terminal transactivation domain [8]. The bZIP domain, similar to that of the Jun/Fos family of oncoprotein, consists of two stretches of basic residues basic regions 1 and 2 (BR1 and BR2) and a leucine zipper [8]. The transactivation domain is characterized by 2.5 proline-rich repeats (PRRs), which contain several SH3-binding motifs [8]. Several studies showed that the attenuated MDV shows some deletions in the BamHI-D and H fragments and has an inserted repeat sequence in the unique long region (UL) of the genome compared to the parent [9]. On the other hand, attenuated strains of MDV1 are not oncogenic although no structural or transcriptional changes have been reported concerning *Meq* gene [10]. Several reports suggested that the number of PRRs and point mutations in PPPP stretches might provide an indication of the isolate pathogenicity [4,11]. As a requirement for the disease control in Saudi Arabia (SA), vaccination with a cell associated modified live CVI988 and herpesvirus of turkey (HVT) strain FC 126 are frequently used in broiler and layers chickens at 1-day old. The vaccination failure and inability of the vaccine to protect chickens against overt clinical signs following field infection may be due to increasing in the virulence of the virus or early exposure [12].

In this study, we aimed to characterize MDVs circulated in the eastern region of SA using polymerase chain reaction (PCR) and genomic sequencing and detect the diversity of the *Meq* gene structure between two oncogenic MDVs from field cases.

## Materials and Methods

### Ethical approval

This study was carried out after the necessary permission of Institutional Animal Ethics committee, King Faisal University, Saudi Arabia.

### Case history and clinical specimens

12-15 weeks old layer chickens from two farms in the eastern region, SA, vaccinated with commercial MDV vaccines (contains cell associated modified live Marek’s Rispens CVI988 strain virus and HVT strain FC 126), were represented to the Avian Clinic, Veterinary Teaching Hospital, King Faisal University, Al-Hassa, SA. Birds showing high mortality (10%) with signs of depression and general weakness. Birds subjected to routine postmortem examination. Samples of liver, spleen, kidneys, and proventriculus were collected aseptically and subjected to molecular detection and characterization of MDV in the Central Biotechnology Laboratory.

### DNA extraction

Total DNA was extracted from up to 25 mg spleen samples as well as commercial live attenuated MDV as a positive control using DNeasy Blood and Tissue Kit (QIAGEN, USA). After complete lysis of the specimens by ATL buffer and proteinase K, absolute ethanol was added then the mixture was transferred to a spin column according to manufacturer’s protocol. Purified DNAs were recovered in 150 µl AE buffer and stored at −20°C for further testing.

### Detection of the *Meq* oncoprotein gene

The extracted DNAs were screened for presence of MDV using HotStartTaq^®^ Plus Master Mix Kit (QIAGEN, USA). 2 µl sample of each purified genomic DNAs was amplified in 20 µl of the final volume of a 2X HotStartTaq Plus Master Mix containing 1.5 mM MgCl_2_, 200 µM of each dNTP, 1 unit HotStartTaq Plus DNA polymerase, and 10 µM of each forward (F:GCACTCTAGAGTGTA AAGAGATGTC TCAG) and reverse (R:TAACTCG AGGAGAAGAAACATGG GGCATAG) primers [13]. Thermo-cycling conditions were enzyme activation and initial denaturation at 95°C for 5 min followed by 35 cycles of 94°C for 30 s, 55°C for 30 s, and 72°C for 30 s and a final extension step at 72°C for 10 min. The amplified PCR products were electrophoresed in 1.5% agarose gel stained with ethidium bromide and documented using ultraviolet gel documentation system (BIORAD).

### Sequencing and construction of phylogenetic tree

*Meq* gene specific bands were excised from the agarose gel, purified using Montàge DNA gel extraction kit (Millipore, USA) and sequenced in an automated ABI 3730 DNA sequencer (Applied Biosystems, USA). The obtained sequence was aligned by the Clustal W method. The obtained nucleotide sequences were compared with MDV sequences available in Genbank by BLAST web tool of the Genbank (Table-1). A phylogenetic tree was constructed using MEGA version 5.20 software.

**Table-1:** MDV reference strains used in construction the phylogenetic tree.

Accession No.	Country	Name	Reference
HM991861	China	MDV/BY/China	[30]
HQ638151	China	MDV/TQ20/CH	[31]
EF546430	China	MDV/GXY2/CH	Unpublished
HQ658624	China	MDV/HLJ/07/II	[7]
HQ658619	China	MDV/LN/08/V	[7]
HQ658627	China	MDV/HLJ/06/I	[7]
AY362712	USA	MDV/617A	[21]
KJ464784	Poland	MDV/12_08 LORF7	Unpublished
KJ464771	Poland	MDV/5_06 LORF7	Unpublished
HQ204815	Poland	MDV/73_08_PL	Unpublished
AY362725	USA	MDV/648A	[21]
AF243438	USA	MDV/Md5	[5]
HM749326	India	MDV/tn-n3	Unpublished
AB638844	Japan	MDV/Tokachi-s1	[28]
KC243264	Iraq	MDV/10A	[11]
KC243266	Iraq	MDV/51C	[11]
KC243266	Iraq	MDV/95E	[11]
EF523775	Australia	MDV/Woodsland1	Unpublished
EF523775	Australia	MDV/FT158	Unpublished
AF493558	China	MDV/648A	Unpublished
AF493555	Netherland	MDV/CVI988	Unpublished
HF546085	China	MDV/HNGS201	[12]
KC161221	Egypt	MDV/Egypt_5	[32]
EF523390	USA	MDV/RB-1B	Unpublished
AF147806	USA	MDV/GA	[22]
EF523774	Australia	MDV/MPF75	Unpublished
JX467678	Egypt	MDV/Egypt_1	[32]
JN808272	India	MDV/ABT/HSR/5253	Unpublished
JN808280	India	MDV/ABT/HSR/7158	Unpublished
KJ464769	Poland	Wroclaw_06 LORF7	Unpublished

MDV=Marek’s disease virus

### Genbank accession number

The obtained *Meq* gene sequences of the detected MDV were submitted to the GenBank database with the accession number (Saudi 01-13; KJ949617 and Saudi 02-13; KJ949618).

## Results

### Clinical examination

Birds necropsy and morphological observations of the visceral organs revealed enlarged liver with rounded edges multiple grayish tumors and the spleen were enlarged with grayish nodules.

### Molecular detection of *Meq* gene

PCR analysis of *Meq* gene ORF specific for MDV serotypes 1 was done to detect MVD in DNA of tested samples. A 1062 bp fragment was detected in sample 1, whereas in sample 2, a smaller fragment was observed (939 bp).

### Sequence and phylogenetic analysis of different *Meq* gene fragments

**Figure-1:**
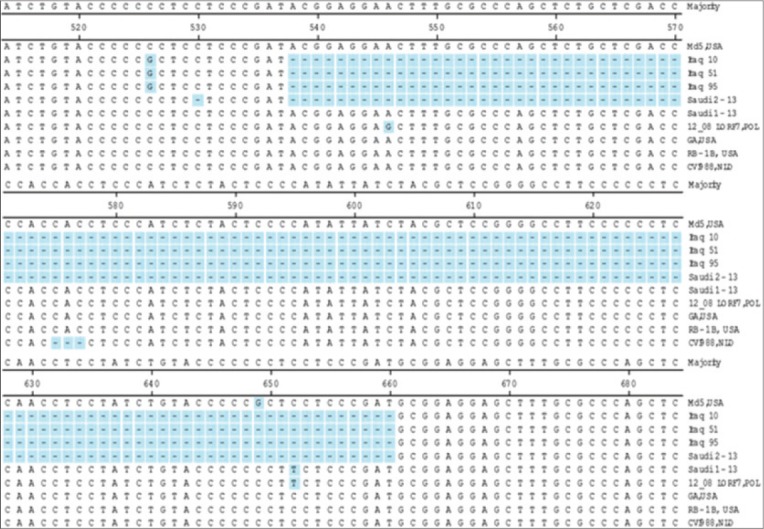
Alignment of *Meq* gene sequences. The nucleotides alignment of *Meq* genes from the strains listed at the right. Unmatched sequences represented by dashes (−). Deletion of nucleotides (538-660) in Saudi 02-13 without frameshifting.

**Figure-2:**
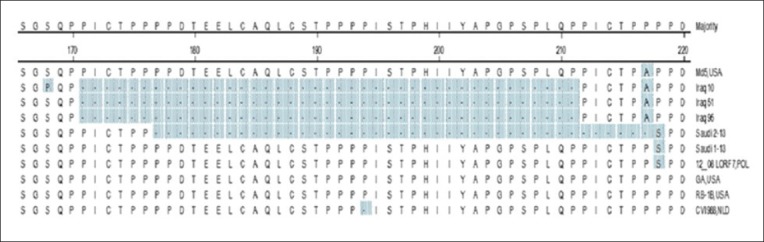
Alignment of *Meq* gene amino acids sequences. The amino acid alignment of *Meq* genes from the strains listed at the right. Unmatched sequences represented by dashes (−). Deletion of amino acids (177-217) in Saudi 02-13 without frameshifting.

Nucleotide gene sequence of the *Meq* gene ORF detected in Saudi 01-13 was 1062 bp encoding for a polypeptide of 339 amino acids, whereas the *S-Meq* ORF of Saudi 02-13 (939 bp) showed deletion of about 123 bp between the nucleotides 538 and 660 of the ORF. The deleted area encoding for a polypeptide 41 amino acids without frameshift (Figures-1 and 2). The deletion site was identified between two PRR regions in C-terminal proline-rich domain. Nucleotide and deduced amino acid sequences of the MDV isolates from SA were highly conserved when compared to MDV strains worldwide (CVI988/NLT, GA/USA, Md5/USA, 12_08LO-RF7/PL, and RB-1B/USA). Three amino acid substitutions: Asp.80Tyr., Cys.110Ser., and Pro.218Ser. were identified in the *Meq* of MDVs isolated from SA and MDV strain from Poland (12_08LO-RF7/POL), in addition, eight amino acids substitutions were identified among the Saudi strains and MDVs from Iraq (Table-2).

**Table-2:** Amino acid substitution of Meq gene protein.

Strain/country	Amino acids position/substitutions												

	Basic region			Leu Zip	Transactivation domain								
		
	71	77	80	110	141	168	194	200	217	218	283	320	328
CVI988/NLD	S	E	D	C	H	S	-	I	P	P	A	I	S
GA/USA	A	K	D	C	H	S	P	I	P	P	A	I	S
Md5/USA	A	K	D	C	H	S	P	I	A	P	V	T	S
Saudi 01-13	A	E	Y	S	P	S	P	I	P	S	A	I	S
Saudi 02-13*	A	E	Y	S	P	S	-	-	-	S	A	I	S
12_08/LORF7/POL	A	E	Y	S	P	S	P	I	P	S	A	I	L
RB-1B/USA	A	K	D	C	H	S	P	I	P	P	A	I	S
Iraq 10*	S	E	D	C	H	P	-	-	A	P	A	I	S
Iraq 51*	S	E	D	C	H	S	-	-	A	P	A	I	S
Iraq 95*	S	E	D	C	H	S	-	-	A	P	A	I	S

* MDVs isolates with SMeq.

MDV=Marek’s disease virus

### Phylogenetic analysis

**Figure-3:**
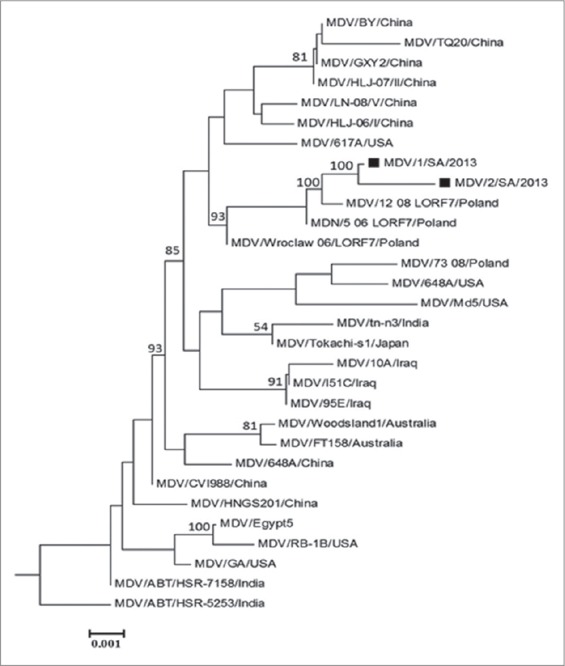
Phylogenetic analysis on *Meq* gene sequences of 2 Saudi Arabian isolates and other 30 references Marek’s disease virus. The phylogenetic tree was constructed using the MEGA version 5.0 by the neighbor-joining method with 1000 bootstrap replicates. Black squares indicate the two isolates from Saudi Arabia.

**Figure-4:**
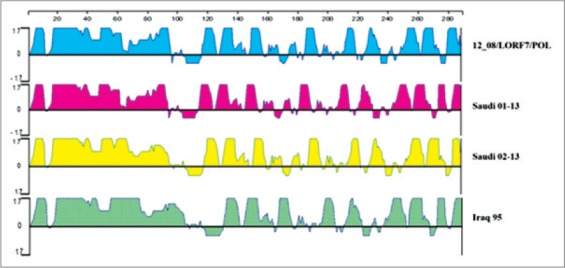
Antigenic index according to Jameson-Wolf for both Saudi Marek’s disease virus, Iraq 95 and 12_08/LORF7/POL. Using Protean analysis DNASTAR Software Package.

The obtained nucleotide sequences of *Meq* gene (Saudi 01-13 and Saudi 02-13) were compared with those of 30 references MDVs summarized in Table-1 for homology analysis using MEGA version 5.2. These 30 reference MDVs representing different regions all over the world. The Saudi 01-13 and Saudi 02-13 had the highest nucleotide homology (99.8% and 99.6%) with 12 08LORF7/PL, respectively (Figure-3). Comparing the antigenic peaks (index) of both Saudi MDVs, Iraq 95 and 12_08/LORF7/POL, the data showed that they are quite similar although the Saudi 02-13 and Iraq 95 showing a deletion of 123 nucleotide representing 41 amino acid which support the hypothesis that the deletion did not cause frame shift (Figure-4).

## Discussion

In the last 40 years, the incidence and interest to MDV has been increasing because of the intensive use of vaccination to control the disease [14]. The virus isolation, genomic sequencing as well as monitoring the oncogenic genes changes are played an important role in the prevention and control of MDV infection in chickens [15]. The polymorphism of *Meq* gene amino acids sequences, as well as point mutations, was found to be correlated to MDV1 virulence [16-18]. The *Meq* gene product is a transcription factor with N-terminal bZIP proteins homologs to Jun/Fos oncoproteins [6].

*Meq* can interact with itself and cellular proteins such as p53 and C-terminal binding protein and can contribute to cellular oncogenesis [19]. Analysis of MDV genome showed several changes including the expansion of the 132 bp direct repeats located in the internal repeat regions flanking the long unique region [20,21]. On the other hand, differences in the *Meq* gene between oncogenic and non-oncogenic MDV1 have been reported as a result of 177 or 180 bp insertion in the *Meq* gene that may postulate as a cause of biological changes results in attenuation of the MDV1 oncogenic strains [16,18,22].

In this study, the main complaint of the owners of two layer flocks aged 12 and 15 weeks old chickens were uneven growth and about 10% mortalities. Following necropsy, there have been found enlargement of the liver and spleen with grayish, yellowish nodules. No lesions were seen on the skin as well as nervous tissues. In addition, gross lesions associated with emaciation were recorded as previously reported [23-26]. Lesions were suggestive for MD [27]. For further diagnosis, samples were tested using conventional PCR [13]. PCR was positive for *Meq* gene of MDVs. Unlikely, predicted the size of the PCR products from Saudi 01-13 and Saudi 02-13 were different. The reason why the two bands are different may be due to genetic diversity (deletion or insertion) of the amplified *Meq* gene as reported by Chang *et al*. [18] and Lee *et al*. [22].

The *Meq* gene of both MDV isolates was compared with five standard MDVs, deletion of 123 bp in the *Meq* ORF (538-660) between two PRRs in the C-terminal proline-rich domain was detected in Saudi 02-13. The deletion did not cause any frameshift in the *Meq* gene ORF. Wajid *et al*. [11] reported a 123 bp deletion in *Meq* gene from Iraq. Surprisingly, the deletion in the *Meq* gene of Iraq strains starts from the same nucleotide positions as Saudi isolates (Figure-1). Whereas, on the corresponding amino acid sequence, the deletion occurred between 2 proline residues ^170^P↓P^171^ while in Saudi isolates the deletion was in PRR ^175^PP↓PP^178^ this may be due to the missed nucleotide at position 530 in the Saudi 02-13 (Figure-2). Previous studies showed that the *Meq* gene is considered the most important molecule in MDV oncogenicity and among the notable finding related to the virulence was distinct diversity and point mutation in the *Meq* proteins [4,18,21-28]. Structural changes in the MDV genome were previously reported including 200-bp deletion in *Bam*HI/L of MDV strain MD11 and 400 bp deletion in the *Bam*HI-A region of MDV strain CVI988 [29]. On the hand, insertion of 177 or 180 bp in the *Meq* gene of CVI988 was reported and does not cause any frameshift in *Meq* gene ORF that may result in attenuation [21,22]. The number of PPPP motif in the PRRs was 4 in the Saudi 01-13 and 2 in the Saudi 02-13; this previously reported as a virulence dependent factor the low number of PRRs is correlated to the high in virulence [4,11]. Both Saudi isolates had point mutations that interrupted extensions of four proline at position 3 ^216^PPPP^219^ to ^216^PPSP^219^ which are a unique substitution in Saudi isolates and 12_08LO-RF7/POL. Comparison of *Meq* gene sequence of the studied strains with 30 reference MDV1 strains revealed that the SA MDV strains clustered with the MDV strains from Europe (12 08LORF7/POL) that may be contributed to the importation of poultry and/or poultry by-products from European countries or due to movement of birds during migration.

## Conclusion

For our knowledge, this is the first comprehensive study describe the incidence of MDV in SA. Both of the detected MDV strains causing lymphomas in layer chickens. Based on the clinical picture and the genomic sequencing both of the MDV isolates found to have characteristics of virulent MDVs although the Saudi 02-13 showed deletion of 123 bp without causing any frameshift. The antigenic index of Saudi MDVs and certain regional and international isolates were quite similar. In Saudi 02-13, the amino acid deletion started at position 177 is due to the short isoform of *Meq*. More concern should be given to the imported poultry and poultry by-products, migratory birds as well as vaccination process to control the MDV infection.

## Authors’ Contributions

MHM and IME: Study design, PCR, Genomic analysis and prepared the manuscript. MA and YA: Collected samples. All authors read and approved the final manuscript.
